# A Qualitative Study Exploring the Management of Medicine Shortages in the Community Pharmacy of Pakistan

**DOI:** 10.3390/ijerph182010665

**Published:** 2021-10-12

**Authors:** Sumaira Omer, Salamat Ali, Sundus Shukar, Ali Hassan Gillani, Yu Fang, Caijun Yang

**Affiliations:** 1 Department of Pharmacy Administration and Clinical Pharmacy, School of Pharmacy, Xi’an Jiaotong University, Xi’an 710061, China; sammaraomer@gmail.com (S.O.); sundushukar@gmail.com (S.S.); hassangillaniali@yahoo.com (A.H.G.); yufang@mail.xjtu.edu.cn (Y.F.); 2 Center for Drug Safety and Policy Research, Xian Jiaotong University, Xi’an 710061, China; 3 Shaanxi Centre for Health Reform and Development Research, Xi’an 710061, China; 4 Research Institute for Drug Safety and Monitoring, Institute of Pharmaceutical Science and Technology, China’s Western Technological Innovation Harbor, Xi’an 710061, China; 5 Department of Pharmacy, Quaid-i-Azam University, Islamabad 45320, Pakistan; salamataligondal@gmail.com

**Keywords:** qualitative interviews, essential medicines, shortages, community setting, Pakistan

## Abstract

Managing medicine shortages consumes ample time of pharmacists worldwide. This study aimed to explore the strategies and resources being utilized by community pharmacists to tackle a typical shortage problem. Qualitative face-to-face interviews were conducted. A total of 31 community pharmacists from three cities (Lahore, Multan, and Dera Ghazi Khan) in Pakistan were sampled, using a purposive approach. All interviews were audio taped, transcribed verbatim, and subjected to thematic analysis. The analysis yielded five broad themes and eighteen subthemes. The themes highlighted (1) the current scenarios of medicine shortages in a community setting, (2) barriers encountered during the shortage management, (3) impacts, (4) corrective actions performed for handling shortages and (4) future interventions. Participants reported that medicine shortages were frequent. Unethical activities such as black marketing, stockpiling, bias distribution and bulk purchasing were the main barriers. With respect to managing shortages, maintaining inventories was the most common proactive approach, while the recommendation of alternative drugs to patients was the most common counteractive approach. Based on the findings, management strategies for current shortages in community pharmacies are insufficient. Shortages would continue unless potential barriers are addressed through proper monitoring of the sale and consumption of drugs, fair distribution, early communication, and collaboration.

## 1. Introduction

The World Health Organization (WHO) has recognized the presence of medicine shortages in both developed and developing countries [1]. In 2017, the WHO reported that almost two billion people have no access to basic medicine, leading to less patient care and costly financial consequences [2]. As shortages become more ubiquitous, pharmacists and other healthcare professionals are also spending more time managing them [3]. This means that shortages are likely to impact the workload and clinical decision-making process of healthcare professionals.

There is a paucity of literature, exploring strategies for shortages management at a community pharmacy. Previous research was more concerned on determining the frequency of medicine shortages at community pharmacies [4,5,6]. According to a Finnish study, 80% of community pharmacies encountered shortages almost daily over the 27-day study period [4]. Similarly, in 2015, an Irish survey identified the number, and duration of shortages in a community setting. This study explored that 65/1232 (5.3%) of dispensed drugs were unavailable and had a shortage duration of 13 days on average [5]. The Pharmaceutical Group of the European Union survey (PGEU) examined medicine shortages at EU level as reducing patient confidence (92%), employee satisfaction (79%) and increasing financial losses (82%) [6].

According to the WHO report, the availability of essential medicines in LMICs is only 35 percent in public institutions and 66 percent in the private sector, and this is also true for Pakistan’s healthcare system [7]. Due to the unavailability of essential medicines in Pakistani public hospitals, doctors force the patient, nearly 67%, to visit community pharmacies. Patients therefore obtain medicines from the private sector instead of from public hospitals [8]. Moreover, patients prefer to buy medicines for minor ailments at community pharmacies to avoid long queues and waiting times in hospitals [9]. Almost 80% of the medicines are provided through these channels [10]. 

The black marketing of medicine is also one of the reasons why Pakistan is facing medicine shortages [11,12]. When drugs become in short supply, they are hoarded and sold to patients at higher prices due to a weak enforcement of the law [13]. Whenever the seasonal demand for certain drugs increases, suppliers stock this product or relocate it to other districts, which eventually exacerbates the shortage situation [11].

Addressing medicine shortages is a difficult task, one that requires advanced professional skills such as extensive pharmaceutical expertise, strong communication and collaboration skills [14,15]. At hospitals, the health care team (physicians, pharmacists and nurses) is involved in managing shortages, while in community pharmacies the pharmacist is the sole decision-maker to manage them. Hence, our study aims to uncover the role of community pharmacists in a typical shortage situation, exploring the strategies and resources used to minimize disruption and continuity of patient care, and identifying opportunities for additional support.

## 2. Materials and Methods

### 2.1. Study Design

A qualitative study was performed based on face-to-face interviews using a semi-structured interview guide. We opted for a qualitative design due to the following reasons. Firstly, the design is flexible and can explore respondents’ attitudes, experiences and intentions [16]. Secondly, it generates a wide range of ideas and opinions that individuals carry out about the issues, as well as disclosing different viewpoints among groups [17]. 

### 2.2. Study Setting

This study was carried out in three major cities (Lahore, Multan, and Dera Ghazi khan) in Punjab, Pakistan’s most populated province. These cities belong to different socio-economic levels, with Lahore having the highest gross domestic product (GDP) and Dera Ghazi Khan having the lowest. Lahore city is regarded as the main centre of health, education, business and culture [18].

### 2.3. Interview Guide Development

The interview-guide was adapted from a similar Canadian-based study, whose research objective is consistent with our thinking [19]. This guide included two sections. The first section focused on the demographic questions related to the community setup, whereas the second section assessed the experience of community pharmacists with the shortages including management strategies, perceived barriers, its impact on the pharmacy staff and future interventions (Supplementary Materials). The reliability of the research was assured by preserving records of interviews. The guide was piloted on two community pharmacists before commencing data collection in the form of interviews.

### 2.4. Participant Enrollment 

Participants were recruited using a purposive method until the saturation of themes. Purposive sampling was based on preconceived ideas about the required characteristics of the sample. Thus, target participants were registered community pharmacists who were fluent in the English language, working a minimum of 8 h daily at the community pharmacy, which is the standard working time for community pharmacists in Pakistan [20]. For the selection of community pharmacies, all major locations within the three cities were covered to make the sample as representative as possible.

Community pharmacists were invited to take part in this study at their workplace. Additional information concerning this study was e-mailed to participants at a reasonable request. Those who agreed to participate were interviewed face-to-face by trained investigators (SO and SA). Data were collected from February to May 2021. The selection process of participants is shown in Table 1.

All interviews were conducted in English, because it is a comprehensive and exact language. It allows participants to say what they want to say without having to argue about the meaning [21]. Moreover, pharmacists can easily communicate in English as it is mainly used as a teaching medium in institutions and universities of Pakistan. Each interview took approximately 20 minutes to complete. Participants were provided with an opportunity to freely express additional opinions and comments. All interviews were audio recorded. The researcher wrote field notes during the interviews and shared the written transcripts with the study participants for comments and corrections.

### 2.5. Qualitative Analysis

All interviews were audio-taped, transcribed verbatim, and then analysed under the standard content analysis framework. In the first step, one researcher (SO) listened to the audio recordings several times and transcribed verbatim. SO also used field notes to confirm the understanding of the transcripts. Relevant words, sentences and phrases indicating the objectives of the study were tagged. Initial inductive codes were generated to divide the data, resulting in the formation of a coding table. When several community pharmacists gave the same answer to a particular question, this was seen as a significant finding. Two researchers (SO and SA) then aggregated the final inductive codes into the significant categories. Themes and sub-themes were developed by grouping together several categories to conceptualize the data. Qualitative data managing software (NVivo version 12 Plus, Australia) was used for analysis. All authors had regular discussions to check their common understanding of emerging themes, where ambiguity existed; the final decision was made by the lead author, YC.

Exemplar quotations from the interviewees are presented in the results to demonstrate the findings. Where appropriate, the expressions or words removed from the exemplary quotations have been replaced with three dots.

### 2.6. Reporting

The Consolidated Criteria for Reporting Qualitative Research (COREQ) checklist was taken into account in reporting methods and results [22].

### 2.7. Ethical Permission 

Ethical approval was obtained from Xi’an Jiaotong University, Health Science Center Biomedical Ethics Committee (reference number: 2021–1192) at date 2 February 2021 to develop this research in Pakistan. Participants’ consent was gained after explaining the goals and objectives of the study. Pharmacists working in community pharmacies were contacted and the researcher explained the intent of this research. Each participant’s identity has been kept confidential by issuing an identification code. Participants were allowed to ask any questions regarding the study and all concerns were addressed candidly. We ensured that all the necessary details were clearly communicated. Finally, participating pharmacists were asked to give their consent prior to the start of the interview. A pressure-free and secure environment was maintained during interviews. Study participants were free to raise concerns, skip a question or even withdraw from the interview without giving reasons, although none chose to leave the interview.

## 3. Results

**Figure 1:**
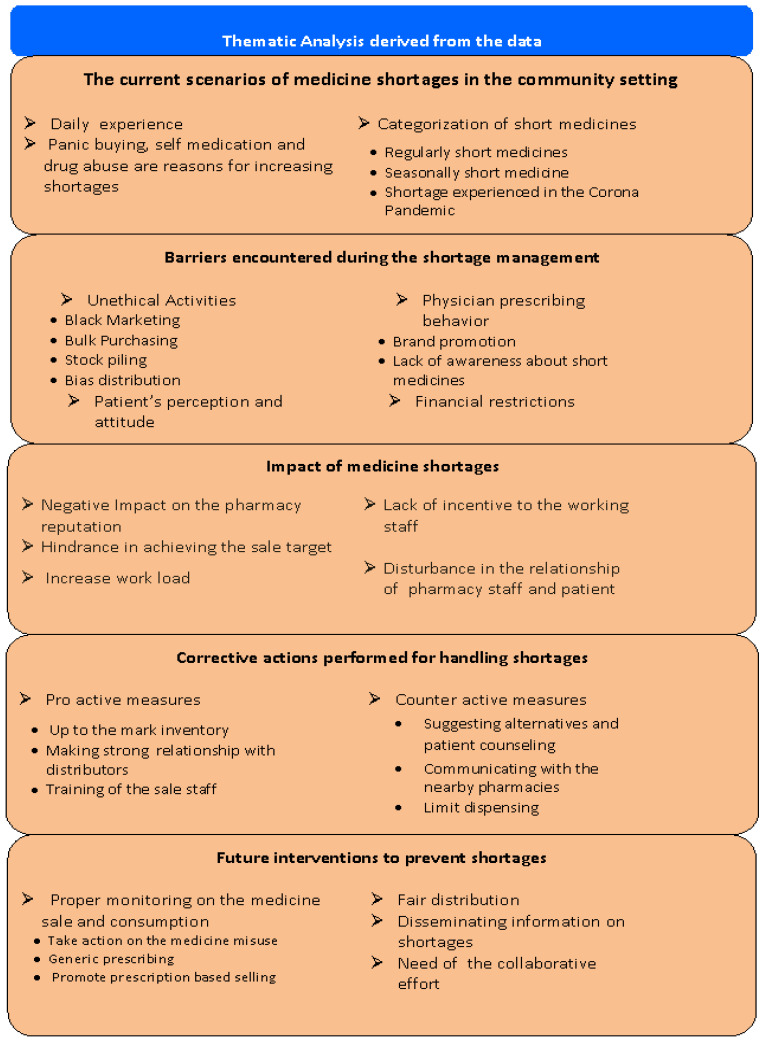
Themes derived from the data.

A total of 31 pharmacists were interviewed. Most were aged between 31 to 40 years. Of those interviewed, the majority were male (25, 80.6%) and employed in the independent community setup (17, 54.8%). The respondents’ characteristics are outlined in the Table 2. The analysis of data yielded five themes, including the current scenarios of medicine shortages in the community setting, barriers encountered during the shortage management, impact of medicine shortages, corrective actions performed for handling shortages and future interventions to prevent shortages. These themes are presented in Figure 1.

### 3.1. Theme 1: The Current Scenarios of Medicine Shortages in the Community Setting

Irrespective of either chain or independent pharmacies, almost all participants claimed to experience medicine shortages in the past year. Essential medicines, such as cardiovascular medicines, anti-diabetic medicines, antibiotics and psychotropic agents were most likely to be in short supply. Participants declared that shortages have surged in recent years, particularly in the COVID-19 pandemic due to panic buying, self-medication and drug abuse. Misinformation and rumours about COVID-19 treatment resulted in panic buying and irrational drug use. Participants reported that there was an increase in demand for multivitamins and calcium in the COVID-19 pandemic. Some medicines were short only in a particular season, for example, the antibiotics used to treat upper respiratory tract infections became short in the winter season, whereas others were continuously in short supply throughout the year, such as psychotropic agents. As an overview, the theme related to the current scenario of medicine shortages in the community setting, along with categories and exemplar quotations, is presented in Table 3. 

### 3.2. Theme 2: Barriers Encountered during the Shortage Management

Community pharmacists reported multiple challenges in managing shortages at various levels. Medicines were often bought from the black market, though it was against the Drug Act of Pakistan. Similarly, stockpiling and bulk purchasing were also noticed in the shortage situation. Beside this, independent pharmacies have confronted discrimination from distributors. Comparing with independent pharmacies, distributors preferred chain-pharmacies for stock delivery, which was unfair to these independent pharmacies.

Patient perception and attitude have hampered the success of shortage management. Even if an alternative of the short-supplied medicine was available, patients refused to buy the alternative as they believe only the medicine written on the prescription was effective. In addition, they could hardly accept the fact that the medicine was short from the supplier end and just blamed the pharmacy staff.

Several participants raised concerns about the physician’s prescribing behaviour. Physicians prescribed medicines with the commodity name, not the generic name, due to the promotion of manufactures. Physicians were unaware about the short-supplied products and prescribed them as well. Financial constraints were also an issue for independent pharmacies. When the short-supplied product became available in the market, it usually came with a high price, which was overwhelming for independent pharmacies. As an overview, the theme related to barriers encountered during the shortage management, along with subthemes, categories, and exemplar quotations, is presented in Table 4.

### 3.3. Theme 3: Impact of Medicine Shortages

Participants reported multiple impacts of shortages on pharmacy business and pharmacy staff. Sales targets were not achieved, and a negative impact on the pharmacy’s reputation was seen. Patients usually lost trust in pharmacies when certain medication was unavailable and refused to buy all if one of the prescribed medicines was out of stock. In this way, shortages have badly affected the reputation and sales of the pharmacy concomitantly.

In times of shortage, due to limited sales and profits, no incentives were provided to workers, and they felt dissatisfied. Additional workloads had been experienced. Interviewees stated that shortages exerted considerable pressure on pharmacists and work staff, taking up time that could have been invested in other important pharmacy activities. It was also reported that the relationship between the pharmacist and patients had been seriously impacted. When some medicine was unavailable, patients blamed pharmacy staff for it. Most of time, patients did not visit again to purchase any medicine. As an overview, the theme related to impact of medicine shortages, along with subthemes, categories, and exemplar quotations is presented in Table 5.

### 3.4. Theme 4: Corrective Actions Performed for Handling Shortages 

Both pharmacies, chain and independent, had relatively similar attitudes towards managing shortages. They used both proactive and counteractive approaches to address the shortages. The most common proactive approach was to maintain inventory up to the mark. For this, purchase orders were placed by keenly observing the prescription trend and previous sales data. A wide range of medicines had been procured. Medicines with high customer demand were purchased in large quantities. In addition, the community pharmacist recorded the missing supplies in a logbook, and sufficient inventory could be purchased when available. Maintaining good relations with distributors and training of the pharmacy were also widely applied proactive approaches.

Suggesting alternative medicine and patient counselling was the most popular counteractive approach. In this context, patients were able to continue treatment without any disruption. Depending on the specific situation, the pharmacist may require a new prescription or suggest a change and get the consent of prescriber. When patients refused the alternative medicine then pharmacists searched the availability of the short medicine at surrounding pharmacies, if they could arrange. Pharmacists have also modified their dispensing practices to deal effectively with the shortages. As an overview, the theme related to corrective actions performed for handling shortages, along with subthemes, categories, and exemplar quotations is presented in Table 6.

It seems that chain pharmacies have an edge over independent pharmacies for handling shortages. Firstly, chain pharmacies have sufficient budgets, and investments to easily prioritize the supply of those medicines that would expect to be in short supply. Secondly, they have a central warehouse to keep the medicine stock. In contrast, independent pharmacies totally rely on distributors to obtain medicine stock, and when they contact distributors to acquire the medicines, they usually cannot obtain the adequate supplies.

### 3.5. Theme 5: Future Interventions to Prevent Shortages

Participants suggested that the sale and use of drugs should be followed up appropriately and regulatory authorities should play a vigilant role and take appropriate measures against drug abuse. Implementing generic prescribing may be one solution to prevent shortages. Prescription-based selling of medicines must be ensured to control the shortages originating from the illegitimate sale of medicines, over consumption, drug misuse and abuse. Participants further elaborated that medicine should be distributed evenly among pharmacies, and biased distribution of medicine to wholesalers and chain pharmacies should be discouraged.

Participants emphasized the importance of communication regarding shortages. Prior notice to health care professionals about shortages would facilitate the implementation of necessary remedial actions earlier. Physicians would not prescribe out of stock medicines, and pharmacists would also have sufficient time to manage inventories.

Addressing shortages through collaboration was also considered as a key recommendation for the health care system. As an overview, the theme related to future interventions to prevent the shortages, along with subthemes, categories, and exemplar quotations is presented in Table 7.

## 4. Discussion

Medicine shortage is an increasing global phenomenon that poses a significant burden to patients and healthcare systems [23]. Although a variety of evidence has characterized the prevalence of drug shortages, little research documented its management practices [24]. This is the first qualitative study performed to examine strategies used to address the shortage within the Pakistani community setting. In this study, five themes and eighteen subthemes were analysed. The themes highlighted the current scenarios of medicine shortages in the community setting, barriers encountered during the shortage management, impacts, corrective action performed for handling shortages and future interventions.

There was a shortage of essential medicines, including cardiovascular medicines, anti-diabetics, antibiotics and psychotropic agents at Pakistani community pharmacies. This result is similar with studies from various other countries such as cardiovascular drugs in Finnish community pharmacies, anti-diabetics in polish pharmacies, and psychotropic agents in a Saudi community setting [4,25,26]. Participants reported a surge in shortages during the pandemic as a result of panic buying, self-medication and substance use. It is also identified that Hydroxycloroquine, multivitamins and the influenza vaccine disappeared from the market during the pandemic. 

Community pharmacists reported multiple difficulties in managing shortages at various levels, including black marketing. Drugs in short supply were unavailable on the open market, but at inflated prices on the black market due to weak enforcement of the law. Mansoor has found in (2016) that the blood pressure medicine, Amplodipine, was sold for 50 times higher than its original price on the black market in Pakistan [27]. Similarly, a Premier Healthcare Alliance survey revealed that grey markets were prevalent for backordered drugs. The average profit margin was 650% above the manufacturer’s sales price, with the highest margins concentrated on the drugs required to treat critical patients [28]. 

Biased medicine distribution has also been a challenge. In Australia, pharmacists employed in independent community settings have also experienced inequitable distribution, and claimed that some chain pharmacies have given priority over independent pharmacies for stock distribution [29].

Patient perception and physician prescribing behaviour were also a challenge to the successful management of shortages. Even though community pharmacists tried to help patients with available substitutes, patients refused to take them since they wanted the same brand that was mentioned in the prescription. Physicians were essentially responsible for developing this mindset in patients because they prescribed medications by commodity name and restricted patients to use any substitute. A recent study by Atif et al. (2019) also found that poor prescribing was one of the barriers to the successful management of shortages in Pakistan [11]. Another gap in prescribing habits was that physicians recommended short medications to patients due to lack of awareness of market status of short supplies.

The shortage situation adversely affected the reputation of pharmacies. Patients did not visit pharmacies with small drug inventories, which had a simultaneous impact on pharmacy sales and credibility. In addition, staff experienced stress with the increased workload to deal with a particular shortage, as it took time to find an appropriate therapeutic alternative and provide relevant information to patients. The Finnish community pharmacists have also reported similar results. Managing shortages at Finnish pharmacies caused increased time burden (52.9%), extra work (21.8%), hasty working environment experience (12.3%), and the median time spent by pharmacy personnel solving drug supply problems was 25 min per week [30].

Although, it is often not possible to anticipate when shortages will happen, the process to address them can be predefined [31]. Therefore, participants took several proactive measures. First, they carefully managed the inventories to ensure that all patients could receive medications for their immediate needs. Second, they developed a strong relationship with distributors. Community pharmacists from Canada also reported that keeping in regular contact with suppliers was a useful strategy to get extra stock before the medicine went on backorder [19]. 

In counteractive measures, the most widely reported approach was to suggest alternative medication to the patient, as it ensures continuous patient care without any disruption. However, pharmacists should be cautious when offering alternative medicine, because it can harm patients by adverse events and medication errors. In 2012, an Associated Press research reported 15 fatalities over a 15-month period due to the complexity of managing medicine shortages [32]. Community pharmacists further elaborated that their efforts to manage shortages are not enough, unless potential barriers are eliminated. Irrational use of medication due to self-medication and drug abuse has exacerbated the shortage problem. Appropriate control over the use of drugs and strict penalties on the illegitimate sale of a prescription-only drug could possibly decrease the level of medicine shortages uplifted by excessive demand. In Pakistan the laws are often developed, but not applied properly. For instance, The Pakistan Drug Law 1976 outlines the manufacturing, distribution and sale of the medicine, but due to its weak enforcement, medicine shortages cannot be managed properly.

Participants also recommended that multi-faceted collaboration between stakeholders is desirable for the successful shortage management from the manufacturer to the distributor and from the physician to the pharmacist, as multiple stakeholders are involved in the complex issue of shortages. However, it is quite difficult to achieve collaboration for shortages management in the Pakistani healthcare system, because physicians and nurses do not accept pharmacists involvement in direct patient care [33]. To avert this, the government need to officially recognize pharmacists as an imperative public health professional, which may reinforce their confidence to act in a global health context [34].

This study had few limitations. Although the sample size was small, the sample size for qualitative research is not designed to represent a large population and focuses instead on the overview of the real situation. The study participants were recruited only from three cities of Pakistan. However, all of the cities were diverse in terms of their community pharmacy setting. Therefore, the findings are expected to be representative of the situation throughout the country.

To solve the problem of medicine shortages, pharmacists, as medication experts, should play an active role. Firstly, the identification of real-time supply chain disruptions and hiring of specialized supply personnel to continuously review drug supply can be useful [35]. Secondly, Pharmacists need to keep close contact with the product manufacturer and distributors to gain insight into the disruption’s origin, projected duration and maintain inventories accordingly [35]. Finally, keeping pace with the growing need for medications and consulting with clinicians to find and offer reasonable alternatives can reduce the impact of drug shortages [36]. 

## 5. Conclusions

This exploratory research identified current strategies used by community pharmacies to manage shortages. Community pharmacists face multiple barriers that have hampered the success of managing shortages. Current management strategies in community pharmacies are insufficient. Shortages would continue unless potential barriers are addressed through the adequate monitoring of the sale and consumption of drugs, fair distribution, early communication and collaboration.

## Figures and Tables

**Table 1:** Participants’ selection for interviews.

Participants’ Recruitment	Number = (*n*)
Participants approached	90
Participants selected based on the inclusion criteria	51
Refused due to specific reason	18
Agreed for participation	33
Final participants at saturation	31

**Table 2:** Participant demographic Profile.

Demographic	Interviewees
Sex (*n*, %)	
Male	25 (80.6)
Female	6 (19.3)
Age (*n*, %)	
20–30	11 (35.5)
31–40	18 (58.1)
>40	2 (6.4)
Experience	7.3 ± (3.8)
Representative of community pharmacy type	
Chain	14 (45.2)
Independent	17 (54.8)

**Table 3:** The Current Scenarios of Medicine Shortages in the Community Setting, subthemes, categories, and exemplar quotations.

Theme 1: The Current Scenarios of Medicine Shortages in the Community Setting	
Subtheme 1: Daily experience Subtheme 2: Panic buying, self medication and drug abuse are reasons for increasing shortages. Subtheme 3: Categorization of the short medicine Regularly short medicines Seasonally short medicines Shortages experienced in the COVID-19 pandemic	“I think the drug shortage is the situation in which our total supply of medicines is insufficient to meet the current or projected demand. I believe that shortage occurs every day in community setup of Pakistan” (CP11) “In my experience, the last year was much more difficult than the previous one, because we have the corona virus in the world beside this if we compare the last five years with the last year, so, we have multiple short medicines in only last year” (CP8) “The main drug which gets short is the Clonazepam comes under the brand name of Rivotril and Magura. All these brands are short from a couple of months and get short every year” (CP3) “We have shortage of antibiotics that is related to the upper respiratory tract infection specifically talking about in the winter season, as you know, the infection prevails in the winter season due to high smog and high fog level so, that medicine in the winter season is always got short from a distributor end” (CP1) “Last year there was a pandemic situation if we take an example; Azithromycin alternates were short and the Hydroxycloroquine tablet was too short, even the patients having arthritis couldn’t get it, just because of the pandemic situation” (CP6)

**Table 4:** Barriers encountered during the shortage management strategies, subthemes, categories, and exemplar quotations.

Theme 2: Barriers Encountered during the Shortage Management	
Sub theme 1: Unethical activities Black marketing Bulk purchasing Stockpiling Bais distribution Subtheme 2: Patient precetion and attitude Subtheme 3:Physician prescribing behavior Brand promtion Lack of awarness about short medicine Subtheme 4: Financial restrictions	“Black marketing is the major offense which we see in a shortage situation. It should not be done”. (CP31) “In the shortage situation, some pharmacies do stockpiling when they get to know that some brands are becoming short in the market, they take bulk quantities. They hoard extra, not affective for the patient and not according to the demand of the patient”. (CP27) “Some distributors are biased for some re known chain pharmacies when they get stock of short medicine they don’t equally distribute their stock to the market”(CP3) “In Pakistan it is a common trend that the physician writes the brands not generics, so they have an edge to promote some brands and when we contact these physicians to write some other brands. They simply stick and refuse” (CP7). “The other barrier which we faced is the financial barrier, because we have to purchase other than our routine and we feel extra overload on ourselves” (CP29)

**Table 5:** Impact of medicine shortages, subthemes, categories, and exemplar quotations.

Theme 3: Impact of Medicine Shortages	
Subtheme 1: Negative impact on the pharmacy reputation Subtheme 2: Hinderance in achieving the sale target Subtheme 3:Increase work load Subtheme 4:Lack of incentive to the working staff Subtheme 5:Distrubance in the relationship of pharmacy staff and patients	“Reputation hits so badly as customers perceived that we don’t have these medicines. So patients would not visit next time to purchase any type of medicine either it is present or not” (CP7) “Drug shortages definitely affected badly our routine operations, it increases workload and it takes a lot of energy in order to overcome such issues.” (CP28) “When customers get bounce due to the shortage of medicines so, we are unable to give incentives and any bonus to the working pharmacy staff”(CP8) “Patients don’t understand any logics and reasons, because they are totally in need of the drug that physicians have prescribed to them” (CP15)

**Table 6:** Corrective actions performed for handling shortages, subthemes, categories, and exemplar quotations.

Theme 4: Corrective Actions Performed for Handling Shortages	
Subtheme 1: Proactive measures Up to the mark inventory Making strong relationship with the distributors Training of the sales staff Subtheme 2: Counter active measures Suggesting alternatives and patient counseling Communicating with the nearbly pharmacy Limit dispensing	“Firstly we keep a sufficient stock of running items. The second practice we have is that we also keep a stock of these products which are not frequently in demand, but we keep it to avoid any rebound of customers”. (CP12) “We have to make relations with the distributions so that they can intimate us properly before any shortages of medicines we can purchase it prior to its shortage” (CP3) “When the medicine is short from the supplier end so either we have to suggest the alternate, or we can ask the patients to contact their doctors to change the medicine” (CP6) “If the patient doesn’t agree to take the alternative so, we then go towards the local purchase. Initially, we try to look for the surrounding pharmacies if they can avail the medicine for us” (CP4) “If we talk about the selling of short medicines, we try to sell it in a limited quantity to maximum customers. For example, if we sell one pack to one customer, in a shortage situation it’s better to sell three blisters to three customers so all people get benefit” (CP13)

**Table 7:** Future interventions to prevent shortages, subthemes, categories, and exemplar quotations.

Theme 5: Future Interventions to Prevent the Shortages	
Subtheme 1: Proper monitoring on the medicine sale and consumption Take action on the medicine misuse Generic prescribing Promote prescription based selling Subtheme 2: Fair Distribution Subtheme 3:Disseminating the information on shortages Subtheme 4:Need of the collaborative effort	“There should be strict actions taken by the regulatory authorities. And not without the recommendation of a doctor, if it’s written on the prescription the right patient will get the medicine” (CP6) “The distributor should distribute medicines first to the retailers and then when they have excess stock then they should deliver to the local market, but the priority should be the retailers”(CP3) “There must be a proper intimation from the supplier end before the medicine shortage. This reporting will be helpful as the medicine suddenly gets short and due to this shortage, we suffer a huge pressure from the consumers’ end because we don’t know the medicine re-availability” (CP7) “Everyone must fulfil their duties to overcome the medicine shortages, because it is not only pharmacist’s task. Purchasers to planners and suppliers to pharmacy managers, everyone is included in it” (CP21)

## Supplementary Materials

The following are available online at https://www.mdpi.com/article/10.3390/ijerph182010665/s1, Supplementary file S1: Interview Guide.
