# Supplementary material for: A Qualitative Study Exploring the Management of Medicine Shortages in the Community Pharmacy of Pakistan
Source: Int J Environ Res Public Health. 2021 Oct 12;18(20):10665. doi: 10.3390/ijerph182010665 (PMC8535806; doi:10.3390/ijerph182010665)
Supplement: Supplementary file 1 [file ijerph-18-10665-s001.zip › ijerph-1346587-supplementary.pdf]

Semi-structured Interview Protocol Questions

- Objective: The study aimed to find the strategies and resources being utilized by community pharmacists to tackle a typical shortage problem
- Consent procedures, including review of study (purpose, objectives, process), permission to audio record, statement regarding removal of information at participant's request, confirmation of understanding
- Thank participant for involvement
- Audio-record verbal consent provided by participant

Part 1: Demographic Question

1. Sex

(a) Male
(b) Female

2. Age

(a) 20–30
(b) 31–40
(c) Above 40

3. Experience (number of years) ________________

4. Representative of community pharmacy type

(a) Chain
(b) Independent

5. Practicing city ________________

Part 2: Medicine Shortage Questions

6. How often do you deal with medicine shortages?

7. How much time do you spend trying to resolve medicine shortages issues?

8. How does dealing with medicine shortages impact you on a day to day basis?

9. Walk us through your strategies/processes that you use when dealing with a medicine shortage situation?

9.1. How do your strategies change in the short term vs long term vs permanent shortage?

9.2. How do your strategies change depending on if the medicine is "critical" to the patient?

9.3 Amongst the strategies you described, which strategy did you find most effective? Why?

10 .What tools or resources did you use when faced with a medicine shortage issue? Was it valuable?

11. What tools or resources would you want to be available when dealing with a medicine shortage?

12. What do you think other pharmacies should or should not do when there is a medicine shortage?

Thank you for participating in this study.
